# Tunable Broadband Terahertz Metamaterial Absorber Based on Vanadium Dioxide and Graphene

**DOI:** 10.3390/mi14091715

**Published:** 2023-08-31

**Authors:** Laifang Zheng, Rui Feng, Huanting Shi, Xuanjing Li

**Affiliations:** 1 Department of Electrical Engineering, Taiyuan Institute of Technology, Taiyuan 030008, China; zhenglf@tit.edu.cn; 2 China Key Laboratory of Micro/Nano Devices and Systems, Ministry of Education, North University of China, Taiyuan 030051, China; shi1826923@163.com (H.S.); 15534081671@163.com (X.L.)

**Keywords:** graphene, metamaterial absorber, terahertz, tunable, vanadium dioxide

## Abstract

We propose a dynamically tunable ultra-broadband terahertz metamaterial absorber, which was based on graphene and vanadium oxide (VO_2_) and numerically demonstrated. The excellent absorption bandwidth almost entirely greater than 90% was as wide as 6.35 THz from 2.30 to 8.65 THz under normal incidence. By changing the conductivity of VO_2_ from 20 S/m to 3 × 10^5^ S/m, the absorption intensity could be dynamically tuned from 6% to 99%. The physical mechanism of the ultra-wideband absorption is discussed based on the interference cancelation, impedance matching theory, and field distributions, and the influences of the structural parameters on absorption are also discussed. According to the symmetric configuration, the absorption spectra of the considered polarizations were very close to each other, resulting in a polarization-insensitive structure. Such a tunable ultra-broadband absorber may have promising potential in the applications of modulating, cloaking, switching, and imaging technology.

## 1. Introduction

The terahertz (THz) wave, whose frequency ranges from 0.1 to 10 THz, has broad application prospects in medical imaging, stealth technology, security inspection, broadband communication, and so on [1,2,3,4]. High-performance devices are essential for effective terahertz wave manipulation in order to achieve these favorable applications. Metamaterial perfect absorbers (MPAs) play an important role in numerous devices owing to their distinctive advantage of ultra-thinness. Following the discovery of the perfect narrow-band microwave absorber by Landy et al. in 2008 [5], many researchers have put forward a variety of metamaterials based on the THz wave absorption device model and have studied its single-band [6], multiband [7], and broadband characteristics [8]. However, the reported MPAs with a metal/insulator/metal structure still have some problems, such as a limited frequency or wavelength, unadjustable absorption performance, and low absorption efficiency, which greatly limit their further practical application. Hence, achieving high-performance tunable ultra-broadband terahertz MPAs has become an important research direction of terahertz technology.

In order to solve the above problems, some new two-dimensional materials have become research breakthroughs. Recently, many MPAs have been proposed to broaden the absorption bandwidth and achieve dynamic tunable characteristics, based on graphene [9,10], MoS_2_ [11,12], LCD [13], and black phosphate [14]. Although these have the advantage of a greater degree of freedom in dynamic tunability, they are difficult to design because of the complex structure of the cell and its array and the shortcomings of incident angle or polarization dependence. VO_2_ is a kind of phase-change material, switching between insulation and metal phase states, and it can be controlled by external stimuli such as heat, with the phase transition time able to be completed in picoseconds. The conductivity difference of VO_2_ between the insulator and metal phase is approximately three orders of magnitude in the THz range, which makes it promising to design innovative devices. Many MPAs with broadband and tunable absorption properties have been reported, based on VO_2_ [15,16,17]. However, there are many problems to be overcome, including their complex structure, narrow bandwidth, and poor absorption effectiveness. Thus, it is worth further exploring a novel broadband terahertz absorber with tunable absorption, improved insensitivity to polarization, and simpler geometry. Graphene is a two-dimensional material that consists of carbon atoms arranged in a planar hexagonal lattice. The Fermi level of graphene can be continuously tuned to change its surface conductivity by chemically doping it or introducing an external bias voltage, thus giving the tunable properties of graphene-based metamaterial absorption structures [18,19,20]. Several multifunctional THz absorbers combining graphene and VO_2_ have been proposed [21,22,23]. However, most terahertz metamaterial absorbers still have some problems, such as a low absorption efficiency, untunable absorption performance, and insensitivity to polarization, which greatly limit their further practical application. Therefore, achieving a high-performance tunable terahertz metamaterial absorber has become an important research direction in the field of terahertz waves.

Inspired by these earlier studies, we proposed a tunable broadband terahertz metamaterial absorber based on vanadium dioxide and graphene. The proposed absorber comprised a periodic array of VO_2_ resonant rings, a graphene layer, an insulator layer, and a metal ground plane. When the VO_2_ was in the metallic state, the bandwidth of the designed absorber was 6.35 THz (2.30–8.65 THz), and the rate of absorption was almost entirely greater than 90%. When the frequency was between 2.30 and 8.65 THz, by controlling the conductivity of VO_2_ from 2 × 10 S/m to 3 × 10^5^ S/m, the absorption peak could be continually tuned from 6% to 99%. Moreover, for both transverse and longitudinal electromagnetic waves, the proposed absorber had an insensitivity to the polarization angle, with considerable incident angle tolerance. In the field of terahertz absorbers, the proposed multifunctional absorber is anticipated to be employed extensively.

## 2. Structure Design and Method

**Figure 1:**
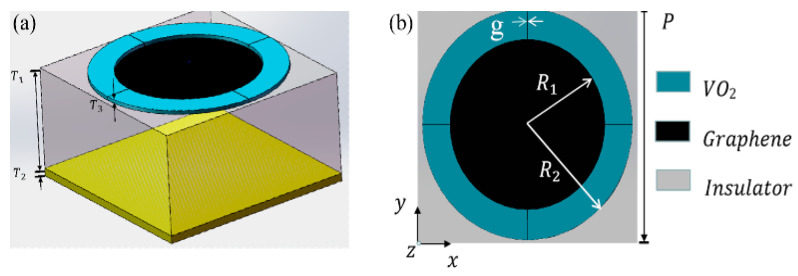
(**a**) 3D schematic of the ultra-broadband terahertz absorber unit cell. (**b**) Top view of the unit cell.

Figure 1 depicts a 3D schematic diagram and the geometric parameters of the designed tunable ultra-broadband terahertz metamaterial absorber. The absorber consisted of four layers, including the Au bottom layer, the insulator layer, the graphene layer, and the VO_2_ layer with a resonant splitting ring pattern. As illustrated in Figure 1, the optimized structural parameters of the absorber unit were Px = 20 μm and Py = 20 μm. The thickness T_2_ and conductivity of the bottom gold layer were 0.2 μm and 4.09 × 10^7^ S/m, respectively [24], and the bottom layer acted as a mirror to ensure the complete reflection of the illuminating terahertz wave, thereby suppressing transmission. The relative permittivity of the insulator layer was 1.96, and it was assumed to be lossless in the simulation [25], with a thickness T_1_ of 9.5 μm. The graphene layer was a single layer, and its thickness was 0 μm. The top VO_2_ layer was composed of a ring with a cross opening, and the thickness T_3_ of the VO_2_ layer was 0.16 μm. The optimized geometric parameters of the splitting ring were R_1_ = 7 μm, R_2_ = 9.5 μm, and g = 0.625 μm. In order to analyze the magnitude response of the designed absorber, the CST microwave studio was used to perform the numerical simulation. We utilized the finite difference time domain (FDTD) method to simulate the structure and obtain the corresponding reflection and transmission coefficients. The PEC-PMC boundary conditions were used in the *x* and *y* directions to simulate the infinite array for normal incidence, while the open boundary was considered along the *z* direction.

The interaction between electromagnetic waves and graphene can be explained by solving Maxwell’s equation. All the calculations were carried out using the computer simulation technology (CST) microwave studio, and 3D numerical results were obtained. Since graphene was modeled as a material with a surface conductivity of $\sigma_{gra}$ in the simulation, the surface conductivity of the graphene could be characterized using the following formulae (i.e., Kubo formula) [26]:

(1) $$\sigma_{gra}=\sigma_{intra}+\sigma_{inter}$$

(2) $$\sigma_{intra}=\frac{2e^{2}k_{B}T}{\pi h^{2}}\frac{i}{\omega +i/\tau }In[2cos\;h(\frac{E_{f}}{2k_{B}T})]$$

(3) $$\sigma_{inter}=\frac{e^{2}}{4h^{2}}[\frac{1}{2}+\frac{1}{\pi }arctan(\frac{h\omega -2E_{f}}{2})\frac{i}{2\pi }In\frac{{\left(h\omega +2E_{f}\right)}^{2}}{{\left(h\omega +2E_{f}\right)}^{2}+{4\left(k_{B}T\right)}^{2}}]$$

where e denotes the charge amount; h is the reduced Planck constant; $k_{B}$ represents the Boltzmann constant; and T, τ, ω, and E_f_ are the ambient temperature (T = 294 K), the electron mobility, the incident light angular frequency, and the graphene Fermi level, respectively.

In accordance with the Pauli repulsion principle, the surface conductivity of the graphene was primarily derived by the intra-band conductivity, while the inter-band conductivity could be ignored. According to Refs. [27,28], Equation (1) can be simplified as:

(4) $$\sigma_{gra}=\frac{e^{2}E_{f}}{\pi h^{2}}\frac{i}{\left(\omega +i/\tau \right)}$$

It can be seen from Equation (4) that the surface conductivity of graphene is not only related to the angular frequency and relaxation time of the incident electromagnetic wave, but also to the Fermi level. Therefore, the surface conductivity of graphene can be adjusted by the relaxation time and the Fermi level. Both chemical doping and changing the bias voltage can be used in practice to attain a different Fermi level for graphene. In this work, the Fermi level E_f_ = 0 eV and carrier relaxation time τ = 0.1 ps were selected for broadband absorption.

In the terahertz range, the Drude model is adopted to describe the optical dielectric constant of VO_2_ [29]:

(5) $${\varepsilon (\omega )}_{{VO}_{2}}=\varepsilon_{\infty }-\frac{\omega_{p}^{2}(\sigma )}{\omega^{2}-i\gamma \omega }$$

where $\varepsilon_{\infty }$, γ, and $\omega_{p}^{2}(\sigma )$ denote the high-frequency relative dielectric constant, the collision frequency, and the plasma frequency related to the conductivity, respectively. In this design, $\varepsilon_{\infty }=9$, $\omega_{p}^{2}(\sigma )=\frac{\sigma }{\sigma_{0}}\omega_{p}^{2}(\sigma_{0})$, $\sigma_{0}=3\times 10^{5}$ S/m, $\omega_{p}^{2}\left(\sigma_{0}\right)=1.45\times 10^{15}$ rad/s, and $\gamma =5.75\times 10^{13}$ rad/s. As a kind of phase change material, the phase change process of VO_2_ is accompanied by substantial alterations in the dielectric constant and electrical conductivity. Through external optical excitation [30], electrical excitation [31], or heating [32], VO_2_ can be altered to a metallic state from an insulating state. Distinct dielectric constants are employed for various phase states during the VO_2_ phase transition from insulator to metal. In the simulation, VO_2_ was insulated, and we set the conductivity of VO_2_ as σ=2×10 S/m in the normal temperature range. By the time the temperature rose to 68 °C, VO_2_ transformed to the metallic state from the insulating state, and the electrical conductivity changed by four orders of magnitude as well. When VO_2_ was in the metallic state, the VO_2_ conductivity was set as $\sigma =3\times 10^{5}$ S/m. By substituting σ into Equation (5), the dielectric constant value of VO_2_ was derived. At room temperature, VO_2_ presented an insulating phase with a conductivity of 20 S/m. When the temperature reached the phase transition temperature, VO_2_ was in the metallic phase, and the conductivity was 2 × 10^5^ S/m.

**Figure 2:**
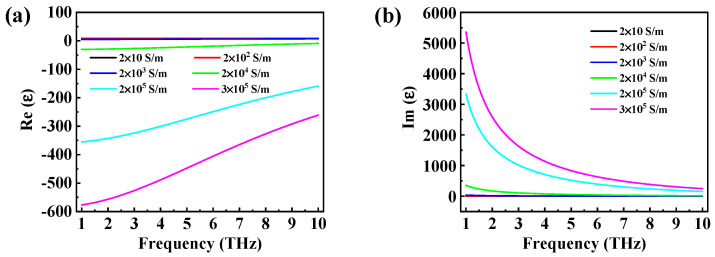
(**a**) The real and (**b**) imaginary parts of the VO_2_ relative dielectric constant for different conductivities.

Figure 2a,b show the evolution of the real part (Re(ε)) and imaginary part (Im(ε)) of the relative dielectric constant of VO_2_ for different values of the conductivity, respectively. It is worth noting that Re(ε) varied from positive to negative with the increase in conductivity. When the state of VO_2_ changed from the protective phase (20 S/m) to the metal phase (300,000 S/m), Im(ε) increased remarkably.

Absorptivity is a crucial parameter for evaluating absorber performance and can be expressed as A(ω) = 1 − R(ω) − T(ω), where T(ω) is the transmission coefficient and R(ω) is the reflection coefficient. Owing to the thickness of the metal film being greater than the penetration depth, T(ω) was close to zero within the operating frequency range. Therefore, A(ω) = 1 − R(ω) could be used to simplify the equation above. The absorption characteristics of this design were estimated by employing the commercial software CST 2018 on the basis of the FDTD method, where periodic boundary conditions are used in the *x* and *y* directions and Floquet ports are used in the *z* direction, and a plane wave was incident on the designed absorbers.

Although numerical simulation could be used to design the proposed device structure, the potential feasible preparation methods were worth exploring. Therefore, a potential fabrication method was proposed. First, a 200 nm thick metallic ground layer was deposited on a glass substrate by magnetron sputtering. A 9.5 μm thick dielectric layer is coated by spin coating. Then, a single layer of graphene was fabricated using chemical vapor deposition and a picosecond laser [33]. Next, 160 nm thick VO_2_ was deposited on the graphene patterned by magnetron sputtering [34]. The ion gel was prepared by dissolving PVDF-HFP in acetone with the aid of a magnetic stirrer for an hour, before adding the EMIM TFSI into the solution and stirring for 24 h. The 100 nm thick ion gel was then spin-coated on the structure and dried in ambient conditions for one hour [35]. Using the above steps, the final sample could be prepared.

## 3. Results and Discussion

**Figure 3:**
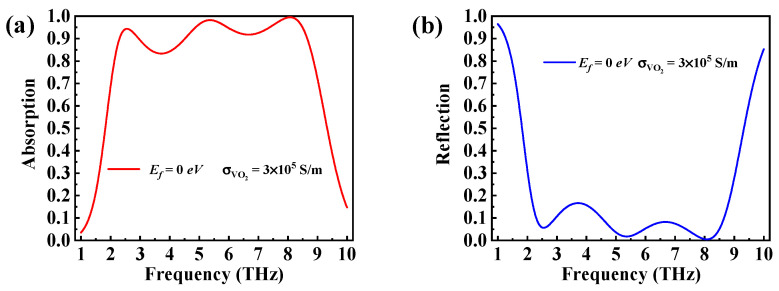
(**a**) Absorption and (**b**) reflection curves when $E_{f}=0\;eV$ and $\sigma_{VO2}=3\times 10^{5}\;S/m$.

When the VO_2_ was in the metal phase state with a conductivity of 3 × 10^5^ S/m, the absorption spectra of the proposed broadband absorber were calculated and shown in Figure 3. The absorption bandwidth with an absorptivity of more than 90% was found to be 6.35 THz (2.30–8.65 THz). The absorption was very high, but it was not uniform. There were certain kinds of oscillation. The cause of these oscillations was graphene film interference. It was observed that two perfect absorption peaks were located at $f_{1}=5.36$ THz and $f_{2}=8.08$ THz.

**Figure 4:**
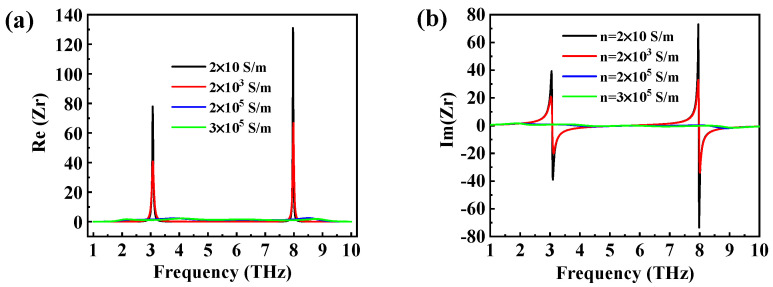
The (**a**) real part and (**b**) imaginary part of the relative impedance in broadband absorption.

Impedance matching, which explains that absorption tends to unity when the effective impedances of the free space and the absorber are matched, could also explain the absorption bandwidth of the metasurface absorber presented in this study. According to the impedance matching theory, the absorption of the absorber under normal incidence is as follows [36]:

(6) $$A(\omega )=1-R(\omega )=1-{\left|\frac{Z(\omega )-Z_{0}}{Z(\omega )+Z_{0}}\right|}^{2}=1-{\left|\frac{Z_{r}(\omega )-1}{Z_{r}(\omega )+1}\right|}^{2}$$

where $Z_{0}=\sqrt{\mu_{0}/\epsilon_{0}}$ and $Z(\omega )=\sqrt{\mu (\omega )/\epsilon (\omega )}$ are the effective impedances of the free space and the absorber, respectively, and $Z_{r}(\omega )=Z(\omega )/Z_{0}$ is the relative impedance between the absorber and the free space. The characterization of the metasurface absorber and the calculation of the relative impedance could be achieved using the scattering parameter inversion technique [23]:

(7) $$Z_{r}(\omega )=\sqrt{\frac{{\left(1+S_{11}(\omega )\right)}^{2}-S_{21}{\left(\omega \right)}^{2}}{{\left(1-S_{11}(\omega )\right)}^{2}-S_{21}{\left(\omega \right)}^{2}}}$$

where S_11_ and S_21_ are scattering parameters, in which the first subscript represents the receiving port and the second subscript denotes the excitation port. The necessary condition for a metamaterial absorber to achieve perfect absorption is that the equivalent impedance of the metamaterial absorber matches the impedance of the free space, so that the incident electromagnetic wave enters the absorber to the maximum extent, and then the reflection reaches the minimum, so as to realize ultra-broadband absorption characteristics. The reflection is minimal and the absorption is close to the unit when the impedance of the absorber equals that of the free space or the equivalent relative impedance is equal to the unit ($Z_{r}=1$). Figure 4 depicts the real and imaginary parts of the relative impedance. As the conductivity of VO_2_ increased, the real part of $Z_{r}$ inclined to 1, the imaginary part inclined to 0, and the absorption bandwidth became wider. When the conductivity of VO_2_ was $3\times 10^{5}$ S/m, the real part approached 1 and the imaginary part approached 0, which meant that the impedance of the proposed absorber almost matched that of the free space. At this time, the reflection of the absorber structure on the incident electromagnetic wave was almost zero, and the maximum loss of the incident terahertz wave was inside the insulation layer, achieving nearly perfect absorption.

**Figure 5:**
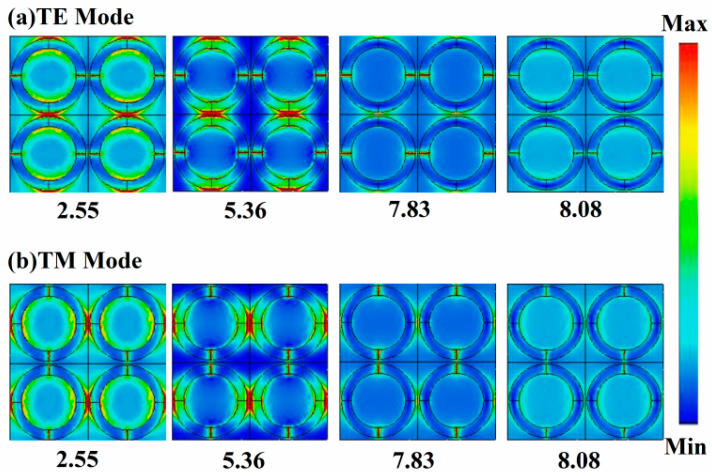
The simulated electric field intensity distributions of the proposed absorber at the frequencies of 2.55 THz, 5.36 THz, 7.83 THz, and 8.08 THz for (**a**) the TE mode and (**b**) the TM mode.

In order to further explain the working mechanism of the broadband absorber, the electric field was investigated at frequencies of 2.55, 5.36, 7.83, and 8.08 THz, respectively, as illustrated in Figure 5. At the frequencies of 2.55, 5.36, 7.83, and 8.08 THz, the absorption of the absorbers was 94.4%, 98.2%, 98.6%, and 99.5%, respectively, and the absorption gradually increased. When the frequency equaled 2.55 and 5.36 THz, the electric field was concentrated between the four parts of the splitting ring, and the VO_2_ resonance was formed between the left and right parts of the splitting rings. When the frequency equaled 7.83 and 8.08 THz, the electric field between the four parts of the splitting ring decreased, and the electric field at the edge of the splitting ring increased. It can be seen that the resonance at a low frequency was mainly caused by the electrical resonance between the parts of the VO_2_ splitting ring, while the resonance at a high frequency was caused by the resonance of a single part of the VO_2_ splitting ring.

**Figure 6:**
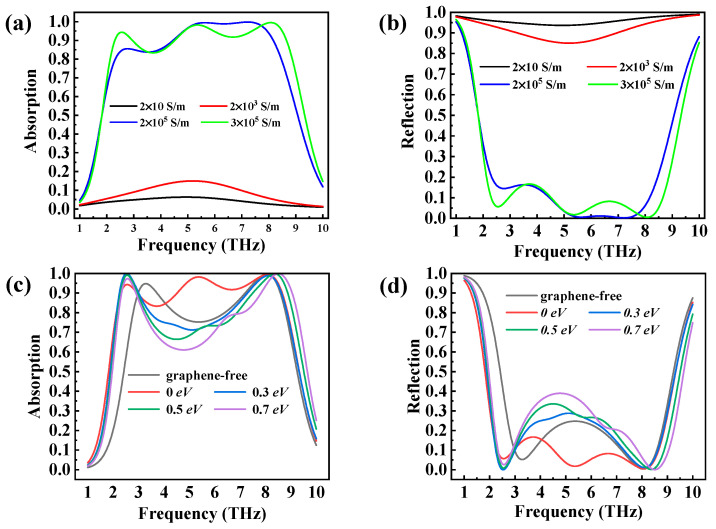
(**a**) Absorption spectra and (**b**) reflection spectra with different conductivities of VO_2_ and a graphene Fermi level of 0 eV, (**c**) Absorption spectra and (**d**) reflection spectra without graphene, with different graphene Fermi levels, and with a VO_2_ conductivity of 3 × 10^5^ S/m.

The absorption of this metasurface absorber could be dynamically regulated by adjusting the VO_2_ conductivity from 2 × 10 S/m to 3 × 10^5^ S/m. Figure 6 describes the VO_2_ simulated absorption spectra with different electrical conductivity values under normal incidence and a graphene Fermi level of 0 eV, as well as the absorption spectra without graphene and with different graphene Fermi levels and a VO_2_ conductivity of 3 × 10^5^ S/m. Thus, the conductivity of VO_2_ and the Fermi level of the graphene had an important effect on the designed absorption system performance, which offered a further degree of flexibility for the implementation of dynamically adjustable ultra-broadband absorbers.

**Figure 7:**
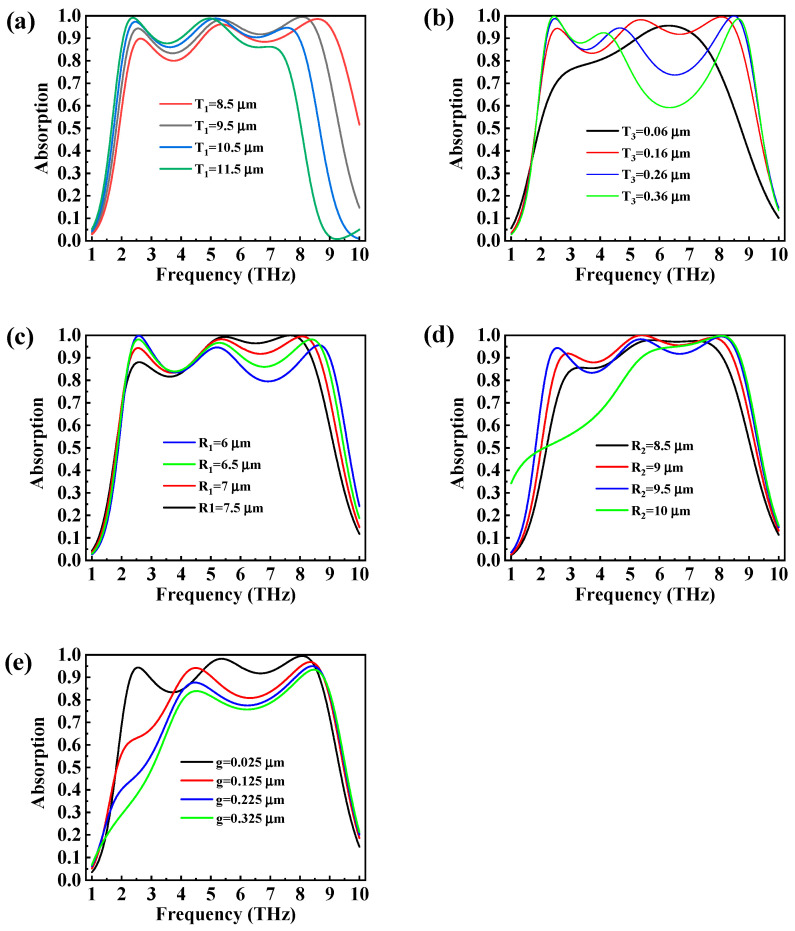
The absorption spectra of the absorber with distinct geometric parameters (**a**) T_1_, (**b**) T_3_, (**c**) R_1_, (**d**) R_2_, and (**e**) g under a normal TE incident wave.

Considering the possible fabrication errors of the metamaterial structure in the actual machining process, we studied in detail the impact of different geometrical parameters on the broadband absorptive properties. Except for the variable parameters, the parameters were fixed at the initial settings for this investigation. When the Fermi level of the graphene was E_f_ = 0 eV and the structural period was 20 μm, the operating frequency and intensity of the broadband and narrowband absorbance could be adjusted by the parameters. Figure 7 depicts the influence of the parameters (such as T_1_, T_3_, R_1_, R_2,_ and g) on the absorption spectrum of the design metasurface absorber.

As shown in Figure 7a, when T_1_ increased from 8.5 μm to 11.5 μm, a remarkable red shift could be observed at a high frequency such that the relative bandwidth reduced. In order to account for this red-shift phenomenon, the propagation phase ($\varphi_{p}$) could be employed, which is denoted by [37]:

(8) $$f=\frac{c\varphi_{p}}{4T_{1}\sqrt{\varepsilon_{r}-{sin}^{2}\alpha }}$$

where T_1_, f, $\varepsilon_{r}$, c, and α are the thickness of insulator layer, the resonance frequency, the permittivity of the insulator layer, the speed of light in free space, and the incident angle, respectively. In this design, the parameters $\varepsilon_{r}=1.96$ and α=0°. $\varphi_{p}$ were considered to be fixed as well. The incident wave was a plane wave, $\varphi_{p}$. It is known that Equation (8) shows an inverse relationship between the resonant frequency and the thickness of the insulator layer. Therefore, the resonance frequency was red-shifted as parameter T_1_ increased.

The absorption spectra of the designed absorber with different thicknesses of the bottom layer are depicted in Figure 7b. When the thickness T_3_ was equal to 0.16 μm, the ultra-broadband and quasi-perfect absorption of the designed absorber was achieved. Therefore, the thickness of T_3_ = 0.16 μm was selected as the optimal geometric parameter. Figure 7c,d depict the absorption performance of the absorber with ring radius R_1_ and R_2_, respectively. When the radius R_1_ varied from 6 to 7.5 μm, there was a significant red shift in the high-frequency absorption. On the contrary, as the radius R_2_ increased from 8.5 to 10 μm, the absorption performance at a low frequency had a remarkable red shift. The LC circuit model could be utilized to elucidate the resonant frequency of the proposed absorber, which is expressed as [37]:

(9) $$f=\frac{1}{2\pi \sqrt{LC/2}}\propto \frac{1}{R}$$

where R, C, and L, are the radius, the total capacitance, and the inductance of the ring, respectively. It can be observed that the frequency in this equation has an inverse relationship with the ring radius. Thus, as the radius of the ring increased, the peak of the absorption moved towards a low frequency.

In Figure 7e, as the gap of the splitting ring increased, the absorption between the two resonant frequencies, shifting from 5 THz to 8.65 THz, decreased significantly. Furthermore, the absorption peak at 2.3 THz reduced with an increase in parameter g. However, the bandwidth showed no distinct change. Based on the above considerations, we finally selected the size of the broadband absorber as T_1_ = 9.5 μm, T_2_ = 0.2 μm, T_3_ = 0.16 μm, R_1_ = 7 μm, R_2_ = 9.5 μm, and g = 0.025 μm.

**Figure 8:**
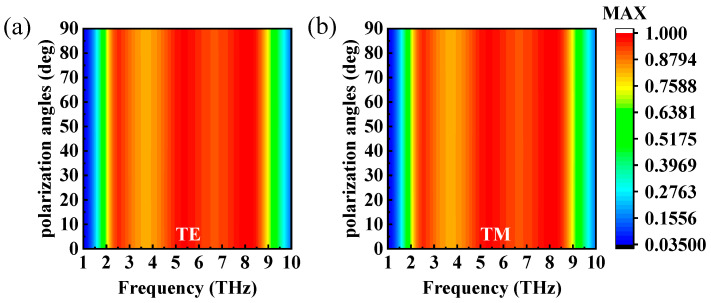
The absorption spectra of the designed absorbers with different polarization angles for (**a**) TE mode and (**b**) TM mode.

In practical applications, it is crucial that the absorber be insensitive to the polarization angle. To investigate the absorption effect of the absorber under oblique incidence, broadband absorption spectra with polarization angles of 0° to 90°, incidence angles of 0° to 90°, and a step size of 10° were simulated in the frequency range from 1 to 10 THz. Figure 8a,b depict the absorption spectra of electromagnetic waves that were incident in the normal direction and had different polarization angles for TM and TE modes. It is clear that the absorption at each frequency point experienced no shift with the change in polarization angle. This polarization-insensitive characterization might have been a result of the symmetrical structure of the designed absorber.

**Figure 9:**
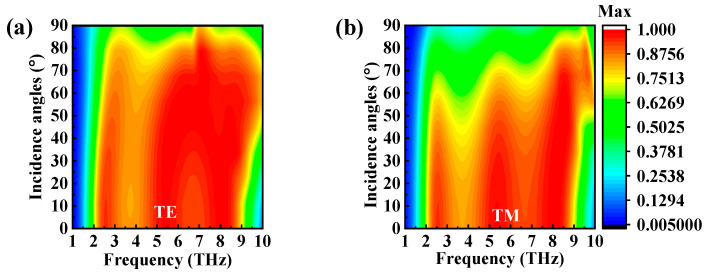
The absorption spectra of the designed absorbers with different incident angles for (**a**) TE mode and (**b**) TM mode.

Figure 9a shows the absorption spectra with various incidence angles for the TE-polarized incident wave. The absorption decreased in the low- and high-frequency absorption bands with the increased incident angle, but a maximum absorption coefficient of 89% could still be achieved at the incident angle of 70°. Figure 9b shows the absorption spectra with various incidence angles for the TM-polarized incident wave. An absorption intensity over 90% could also be obtained until the incident angle rose to 60°. But the absorption bandwidth was greatly affected by the incident angle, since the tangential component of the electric field parallel to the *x* axis of the TM-polarized incident wave decreased with the increase in the angle of incidence. In addition, both polarization situations showed a blue shift with a rising incidence angle, which was caused by the parasitic resonance on the absorber surface at high incidence angles [38].

Terahertz metamaterial absorbers based on VO_2_ and graphene have attracted more and more attention. To illustrate the advantages of the absorbers designed in this paper, we compared their broadband absorption characteristics with those of other published papers in Table 1. The design of the absorber layer number, broadband performance, and adjustable range had good performance.

## 4. Conclusions

In summary, we designed a broadband tunable THz metamaterial absorber using VO_2_ and graphene that could achieve switching performance with nearly perfect broadband absorption. It was demonstrated that the proposed broadband absorber could achieve nearly perfect absorption from 2.30 to 8.65 THz in both numerical simulations and theoretical calculations. In addition, the proposed broadband absorber could be flexibly adjustable, and the absorption amplitude could be continuously increased from 6% to 99% by changing the conductivity of VO_2_. Due to absorbing devices having a tuning range width and polarization insensitivity, etc., they have good application prospects in such areas as tunable filters, sensors, and switches.

## Figures and Tables

**Table 1:** Comparison of broadband absorption performance between different absorbers.

References	Constitutive Materials	Number of Layers	Absorption Bandwidth (THz)	Angular Stability	Polarization Insensitive
[39]	Graphene and VO_2_	7	1.6 (0.8–2.4)	55	Yes
[40]	Graphene and VO_2_	6	1.3 (1.05–2.35)	50	Yes
[41]	VO_2_	3	3.3 (2.34–5.64)	55	Yes
[42]	Graphene and VO_2_	3	1.03 (1–2.03)	50	No
This work	Graphene and VO_2_	4	6.35 (2.30–8.65)	50	Yes

## Data Availability

The data that support the findings of this study are available from the corresponding author upon reasonable request.
